# Updates and future perspectives on neuropsychiatric symptoms in Alzheimer's disease

**DOI:** 10.1002/alz.70079

**Published:** 2025-03-27

**Authors:** Myuri Ruthirakuhan, Dylan X. Guan, Moyra Mortby, Jennifer Gatchel, Ganesh M. Babulal

**Affiliations:** ^1^ Hurvitz Brain Sciences Research Program Sunnybrook Research Institute Toronto Ontario Canada; ^2^ Cummings School of Medicine, and Hotchkiss Brain Institute University of Calgary Calgary Alberta Canada; ^3^ UNSW Ageing Futures Institute University of New South Wales Kensington New South Wales Australia; ^4^ Department of Psychiatry Massachusetts General Hospital Boston Massachusetts USA; ^5^ Department of Neurology Washington University School of Medicine St. Louis Missouri USA; ^6^ Institute of Public Health Washington University St. Louis Missouri USA

**Keywords:** Alzheimer's disease, biomarkers, diagnosis, neuropsychiatric symptoms, treatments

## Abstract

Neuropsychiatric symptoms (NPS) are common throughout the Alzheimer's disease (AD) continuum and profoundly affect patients, caregivers, and health‐care systems. This review synthesizes key research presented in the 2022 and 2023 Alzheimer's Association International Society to Advance Alzheimer's Research and Treatment Neuropsychiatric Syndromes–Professional Interest Area (NPS‐PIA) Year‐In‐Reviews, emphasizing six critical areas: (1) diversity and disparities, (2) diagnostic frameworks, (3) neurobiology of NPS, (4) NPS as a disease marker, (5) the impact of COVID‐19, and (6) interventions. NPS accelerates AD progression, increases functional decline, diminishes quality of life, and heightens caregiver burden and institutionalization rates. Current treatments primarily rely on psychotropics, which offer limited efficacy and raise safety concerns. This review aims to inform clinicians and researchers about recent NPS advancements while identifying gaps for future studies to improve outcomes for individuals with AD.

**Highlights:**

- Research in Alzheimer's disease–related neuropsychiatric symptoms has rapidly increased, indicating heightened interest.
- Key areas include: diversity, diagnostics, markers, COVID‐19 impact, and treatments.
- A road map for future studies, based on the key areas of research, is provided.
- This road map includes considerations to improve study applicability and validity.

## 1 BACKGROUND

The field of neuropsychiatric symptoms (NPS) in Alzheimer's disease (AD) is rapidly evolving, with the yearly publication rate in this field more than double that of previous years over the past decade. This highlights the commitment from researchers and clinicians in this field to improve AD‐related outcomes among patients with NPS.

Despite the growing body of literature, there remains a critical need for an up‐to‐date synthesis of recent advances in this area, as it can be challenging to stay current with the influx of the latest evidence. This paper addresses that gap by providing a comprehensive review of NPS research covered in Year‐In‐Reviews 2022/2023 hosted by the International Society to Advance Alzheimer's Research and Treatment (ISTAART) Neuropsychiatric Syndromes–Professional Interest Area (NPS‐PIA) group, focusing on six key areas that have emerged as priorities in the field: (1) diversity and disparities, (2) diagnostic frameworks, (3) neurobiology of NPS, (4) the investigation of NPS as non‐cognitive disease markers, (5) impact of COVID‐19, and (6) non‐pharmacological and pharmacological interventions. This effort aims to offer a valuable resource for clinicians and researchers, highlighting a synthesis of recent advancements and providing a road map for future studies in this crucial aspect of AD care.

NPS are non‐cognitive behavioral symptoms, such as apathy, anxiety, depression, agitation, and psychosis, that are prevalent throughout the AD continuum and increase in severity with the progression from disease onset. Population‐based studies report that up to 50% of individuals with mild cognitive impairment (MCI)^1^ and up to 97% of individuals with prodromal AD have at least one NPS,^2^ supporting its presence as a core feature of AD. In addition to its prevalence in the AD continuum, acknowledging the negative implications posed by the presence of NPS is essential, contextualizing the research and clinical work being completed in this field.

Though the presence of NPS in cognitively normal individuals is one of the earliest signs of neurodegenerative disease, it is often underdiagnosed and difficult to treat, which consequently leads to widespread negative outcomes for patients, caregivers, and society.^3^ For patients, the presence of NPS has been associated with faster progression to AD dementia,^4^ greater functional decline, reduced quality of life,^5^ and increased mortality risk.^6^ For caregivers, NPS have been associated with reduced quality of life, stress, and increased caregiver burden.^7^ The presence of NPS has also been associated with increased socioeconomic burden as they are often difficult behaviors to manage, their outcomes are a struggle for families and caregivers, and are they increasingly prevalent in the more severe stages of AD, resulting in increased medical costs and rates of institutionalization.^8^

Despite the widespread negative impacts of NPS, their presence is often underdiagnosed in the clinical‐care setting due to lack of awareness, inaccuracies in self‐reporting, and lack of understanding of diagnostic tools, leading to the undertreatment of NPS and consequently poor outcomes. When NPS are appropriately identified, they are often treated off label by psychotropic medications that have demonstrated only modest efficacy and have high‐risk safety profiles.^9^ As such, efforts aimed at improving the diagnostics and management of NPS and identifying novel targets for therapeutic intervention have been clinical and research priorities.

Given the increased interest in identifying, treating, and discovering novel targets for the treatment of NPS, this paper aims to provide a comprehensive review of recent advances in key areas of research focused on NPS. Through this perspective review, we also highlight important considerations and identify key areas of research for future studies.

**RESEARCH‐IN‐CONTEXT**

1. **Systematic review**: The authors reviewed the literature on neuropsychiatric symptoms (NPS) and mild behavioral impairment in Alzheimer's disease for the 2022 and 2023 Year In Reviews hosted by Neuropsychiatric Syndromes Professional Interest Area of the International Society to Advance Alzheimer's Disease and Treatment (ISTAART). Traditional sources were utilized (e,g,, PubMed).
2. **Interpretation**: Key areas of research form 2022/2023 in neuropsychiatric research were identified, which included: 1) diversity and disparities, 2) diagnostic frameworks, 3) neurobiology of NPS, 4) the investigation of NPS as a non‐cognitive disease marker, 5) the impact of COVID‐19, and 6) nonpharmacological and pharmacological interventions. A road map based on knowledge gaps to further research was provided.
3. **Future directions**: Important areas of future research include an in‐depth investigation of health‐related disparities in NPS, incorporation of diagnostic criteria in research, validation of non‐cognitive markers, and trials of nonpharmacological and pharmacological interventions utilizing appropriate diagnostic tools.

### 1.1 Neuropsychiatric symptoms versus mild behavioral impairment

Understanding the differences between NPS and mild behavioral impairment (MBI) is critical to the interpretation of findings. Each construct differs by definition, method of assessment, and duration of symptoms. These differences are outlined in Table 1.

**TABLE 1:** Neuropsychiatric symptoms versus mild behavioral impairment.

	Definition	Symptoms (NPS)/domains (MBI)	Scales/weeks of assessment
**Neuropsychiatric symptoms**	Multidimensional behavioral symptoms ranging from mild to severe, and present across the AD spectrum.	Delusions Hallucinations Agitation Depression Elation Anxiety Apathy Apathy Disinhibition Irritability Sleep Appetite changes	Neuropsychiatric Inventory (Questionnaire/Clinician/Nursing‐Home versions)–4 weeks Geriatric Depression Scale– 4 weeks Cohen Mansfield Agitation Inventory–4 weeks Beck's Depression Inventory–2 weeks Beck's Anxiety Inventory–2 weeks
**Mild behavioral impairment**	Emergence of NPS at ≥ 50 years, persist for at least 6 months, and occur prior to the onset of dementia (normal cognition, subjective cognitive impairment, mild cognitive impairment). MBI symptoms are separate from longstanding patterns of behavior/personality, and are not accounted for by past/present psychiatric disorders.	Decreased motivation Emotional dysregulation Impulse dyscontrol Social Inappropriateness Psychosis	Mild Behavioral Impairment Checklist

Abbreviations: AD, Alzheimer's disease; MBI, mild behavioral impairment; NPS, neuropsychiatric symptoms.

As large datasets have collected data prior to the creation of the MBI checklist, several studies have investigated the association between MBI and various AD‐related outcomes by operationalizing a definition of the MBI using the Neuropsychiatric Inventory Questionnaire (NPI‐Q) and its derivatives^10^: decreased drive/motivation (apathy), emotional dysregulation (depression, anxiety, and elation), impulse dyscontrol (agitation/aggression, irritability, aberrant motor behavior), social inappropriateness (disinhibition), and psychosis (delusions and hallucinations). As the neurovegetative symptoms of appetite and sleep disturbances do not map discretely onto the ISTAART–Alzheimer's Association (AA) MBI criteria,^11^ they are omitted by studies that operationalize the definition of MBI using the NPI. Most studies that have used the NPI‐Q to define the presence of MBI as having symptoms present at two consecutive visits to reflect the “persistent” presence of emergent NPS,^12^, ^13^ though other case operational definitions have been proposed depending on the study design and objective.^14^

## 2 RESULTS

### 2.1 Diversity and inclusion

#### 2.1.1 Race/ethnicity

Dementia prevalence is rapidly rising, with 153 million cases projected by 2050. Two thirds of those cases will be in low‐ and middle‐income countries (LMICs). LMICs are unprepared to handle the surge in dementia with its multidimensional impact on disability, caregiver and financial burden, and disease stigma. NPS are often the last indicators to be addressed in a context with limited resources.^15^

Epidemiological and population‐based studies have consistently demonstrated that NPS are risk factors for dementia in individuals with normal cognition (NC) and MCI.^16^, ^17^, ^18^ However, those studies are primarily based on cohorts recruited in high‐income countries like Canada, the United States, and the United Kingdom, raising concerns about generalizability in more diverse populations.^19^ For example, as ethnic minorities have demonstrated higher rates of cognitive impairment, dementia, and AD compared to individuals who are White, it is likely that the impact of NPS on AD‐related outcomes differs among ethnic groups. Few studies have been published on the comparative risk of NPS on AD between different races/ethnicities, highlighting the need to investigate pathways that reinforce health disparities.

In a population‐based cohort study of 8168 NC individuals from the National Alzheimer's Coordinating Center (NACC) dataset, Babulal et al. reported that the incidence of depression was lower in Black Americans (BAs) compared to non‐Hispanic Whites (nHWs), while there were no other between‐group differences.^19^ Furthermore, BAs and Asians reported lower rates of antidepressant use compared to nHWs. Interestingly, while Hispanics and Asians demonstrated the greatest risk of cognitive impairment compared to nHWs, and depression independently predicted the risk of progression to cognitive impairment, there was no significant statistical interaction between depression and ethnoracial group. Another recent paper from the same group using the NACC cohort with 6958 NC adults demonstrated that relative to persons identifying as nHW, progression to incident cognitive impairment was higher for BA participants endorsing NPS, followed by Hispanic and Asian participants.^20^ Finally, a study in 4984 participants with NC or MCI from the NACC cohort reported higher dementia incidence in BA participants with MBI affective dysregulation, compared to White participants.^21^ Together, those findings demonstrate that the relationship between NPS and ethnoracial groups is complex and the potential role of differential effects on dementia risk.

#### 2.1.2 Sex

Emerging evidence supports that biological sex is an important yet understudied consideration that impacts AD risk. Women have a higher lifetime risk of AD and are more susceptible to related pathology and risk factors like NPS.^22^ For instance, women face double the risk of depression compared to men,^23^ which raises their AD risk by 70%.^24^ Identifying sex‐specific differences in NPS prevalence and severity is essential for understanding their distinct impact on AD risk. Although many studies account for sex as a covariate, few explore whether associations between NPS and AD differ by sex. A more comprehensive approach to studying sex differences in NPS would deepen understanding of the underlying biological factors.

In a comprehensive systematic review and meta‐analysis of 62 studies with 21,554 participants with AD, Eikelboom et al. reported the presence of delusions, psychotic symptoms, aberrant motor behaviors, and depression was greater in females compared to males. Furthermore, females had greater severity of delusions, aberrant motor behaviors, and depression was greater in females, while males had greater severity of apathy.^25^ These differences suggest that the relationship between AD and NPS may be moderated by sex, with varying implications on disease progression. Tremblay et al. found that males with clinicopathologically confirmed AD experienced a greater increase in depressive symptoms compared to females, compared to individuals with NC.^26^ Similarly, Liampas et al. reported that the association between apathy, depression, agitation, and incident AD dementia was moderated by sex. Specifically, apathy and agitation were more strongly associated with AD in males compared to females, whereas depression was more weakly associated with AD in females relative to males.^27^ Kociolek et al. reported that sex modified the association between depressed mood and dependence in a multi‐ethnic sample of community‐dwelling older persons with AD; depressed mood was associated with a greater increase in dependence over time in males but not in females.^28^ Collectively, these findings highlight that there are sex differences in NPS prevalence and severity that may inform the need for personalized approaches to NPS management in AD.

Using data from 8181 elderly adults from the online PROTECT UK study, Wolfova et al. investigated sex differences in the association between MBI and cognitive performance and the rate of cognitive decline in a dementia‐free cohort.^29^ Males had higher rates of decreased motivation, impulse dyscontrol, and social inappropriateness, while emotional dysregulation was more prevalent in females. MBI symptoms were more strongly associated with cognitive decline in males, except for emotional dysregulation, which was associated with cognitive decline in verbal reasoning in females. Those findings highlight that the presence of MBI may have a greater impact on cognitive outcomes in males compared to females. Three studies investigated sex differences in the association between the apolipoprotein E (*APOE*) ε4 allele presence and NPS.^30^ *APOE* ε4 allele presence is a well established genetic risk factor of AD,^31^ with clinical and biological studies supporting female‐specific ε4 effects on cognitive outcomes and AD risk.^32^ Leveraging data from the PACT‐MD study, Dissanayake et al. reported that sex modified the association between ε4 presence and NPS burden. Furthermore, females with two ε4 alleles were more likely to have greater NPS burden in patients with MCI and AD, compared to females with two ε3 alleles. In a cohort of individuals with AD and Parkinson's disease from the Ontario Neurodegenerative Disease Research Institute dataset, Valcic et al. reported that *APOE* ε4/ε4 presence in females was associated with the presence of delusions, while this association was not reported in females with one ε4 allele, and not in males. In neuropathologically confirmed AD patients with Lewy body pathology from the NACC dataset, female ε4 allele carriers were more likely to have psychosis compared to female ε4 non‐carriers, and this relationship was stronger in ε4 homozygotes compared to ε4 heterozygotes.^33^ Those studies suggest that the relationship between *APOE* ε4 allele presence and NPS is moderated by sex, supporting the female‐specific ε4 effects on AD risk reported in previous studies.

### 2.2 Diagnostic frameworks

Developing diagnostic frameworks for NPS is crucial for defining, identifying, and classifying symptoms, with applications in clinical practice, epidemiology, and trials. These frameworks support the investigation of NPS neurobiology, subsequently confirming target engagement, and aiding patient enrichment in clinical trials, which improves diagnostics and symptom monitoring, and identifies those at risk of AD. Updated diagnostic criteria for psychosis,^34^, ^35^ apathy,^36^, ^37^ agitation,^38^ and depression^39^ have been developed through integrating findings from clinical, biomarker, and pharmacological studies, and consensus among key opinion leaders from the NPS‐PIA, International Psychogeriatric Association (IPA), and the International Society for CNS of Clinical Trials and Methodology. Similarly, in 2016, the MBI criteria were established to identify individuals at increased dementia risk, regardless of cognitive symptoms, through a rigorous, iterative process based on NPS and MCI research.^11^

Three comprehensive reviews critically evaluated recent advances in psychosis and apathy to provide evidence‐based suggestions for clinical and biomarker studies.^40^, ^41^, ^42^ With regard to psychosis, subtypes of psychotic symptoms (persecutory vs. misidentification delusions or delusions vs. hallucinations) demonstrated differences in trajectories of disease progression and underlying neurobiology.^40^, ^41^ As such, subtypes of psychotic symptoms may warrant different non‐pharmacological and pharmacological treatment strategies. An updated review on apathy iterates its presence as a prodromal feature of AD.^43^ This is supported by biomarker studies, which report that apathy in the early stages of AD is associated with a higher global amyloid burden, while apathy in the later stages of AD is associated with frontal region dysfunction and elevated tau burden.

In 2023, the IPA formalized a consensus clinical and research definition for agitation in cognitive disorders.^44^ According to this framework, symptoms of agitation fall under three categories: verbal aggression, physical aggression, and excessive motor activity. To meet IPA criteria for agitation, patients must meet criteria for (1) presence of cognitive impairment or dementia syndrome; (2) displays of verbal aggression, physical aggression, and/or excessive motor activity; (3) agitated/aggressive behaviors which are deemed clinically significant; and (4) symptoms not attributable to another psychiatric or medical condition.

### 2.3 Associations with non‐cognitive dementia markers

The high prevalence and impact of AD demand accurate, cost‐effective tools for early detection, crucial for timely intervention. Although early cognitive decline often precedes AD, only 10% to 15% of individuals with MCI progress to dementia annually, while approximately one third revert to NC or remain stable.^45^, ^46^ The 5th Canadian Consensus Conference on Diagnosis and Treatment of Dementia identified sensory changes, motor dysfunction, sleep disturbances, behavioral issues, and frailty as risk factors for dementia.^47^ Addressing these non‐cognitive markers early could reduce dementia risk and alleviate burdens on patients and caregivers. Population studies recognize NPS as predictors of AD but often fail to differentiate between NPS from AD and non‐AD origins^18^, ^48^, ^49^ The ISTAART‐AA MBI criteria were defined in 2016 to identify NPS that are more likely to be a disease marker by purposefully proposing criteria that exclude non‐emergent NPS, non‐persistent NPS, and NPS with defined non‐AD etiology.^11^

In a study with 739 individuals with MCI, McGirr et al. investigated the risk of progression to dementia, stable MCI, or reversion to NC in individuals who had no persistent NPS, NPS but not MBI, and MBI.^50^ While individuals with MBI were less likely to revert back to NC, this difference was not significant in individuals with NPS but not MBI. In 3932 individuals with NC or MCI, Vellone et al. reported that MBI apathy was associated with progression to dementia and that this risk was greatest in those who were *APOE* ε4 carriers.^51^ In 11,372 individuals with NC, Ruthirakuhan et al. investigated the presence of MBI and its domains on progression to clinically diagnosed and neuropathology confirmed AD.^4^ The presence of MBI predicted progression to clinically diagnosed and neuropathology confirmed AD. Furthermore, each MBI domain was associated with an increased risk of progression to incident AD, with psychosis demonstrating the greatest effect. Collectively, these studies support the validity of MBI as a neurobehavioral syndrome that can be used in dementia prognostication.

Three studies used data from the Comprehensive Assessment of Neurodegeneration and Dementia (COMPASS‐ND) study to investigate the association between MBI and frailty,^52^ gait,^53^ and hearing impairment.^54^ In 219 non‐dementia individuals (NC and MCI), the presence of MBI and its domains were associated with greater frailty. Furthermore, the severity of MBI was associated with the severity of frailty in a sex‐dependent manner such that the effect was greater in males compared to females.^52^ In 193 non‐dementia individuals (NC, subjective cognitive decline, and MCI), greater MBI severity was associated with lower gait speed as assessed by dual‐task gait cost.^53^ Finally, in 219 non‐dementia individuals, greater hearing impairment was associated with greater severity in global MBI, MBI–apathy, and MBI–affective symptoms.^54^ A more recent study further demonstrated a longitudinally bidirectional association between MBI and hearing loss.^54^ The findings of those studies suggest that the incorporation of assessments of MBI could be valuable in the clinic to identify individuals at risk of developing dementia. Second, in research, incorporating assessments of MBI could enhance patient enrichment for clinical trials with a disease‐modifying agent. Finally, the associations between MBI and non‐cognitive markers suggest that treating MBI could have positive benefits on frailty, motor function, and hearing impairment. Similarly, improving frailty, motor function, and hearing impairment could have benefits for MBI.

### 2.4 Biomarkers of NPS

Clinical and population‐based studies consistently show that both NPS and MBI are risk factors for AD. Investigating the neurobiological mechanisms behind their manifestation and severity is crucial to establish their diagnostic and prognostic value as AD markers, which could inform treatment strategies for risk mitigation. Biomarkers, whether fluid or imaging, each offer unique advantages in neuropsychiatric research. Fluid markers, more accessible and cost effective for elderly populations, complement imaging markers, which reveal structural brain changes. Understanding how both modalities can predict NPS and MBI is essential for developing targeted treatments and mitigating cognitive decline and dementia risk, with significant clinical and research implications.

#### 2.4.1 Fluid markers

Leveraging data from 784 community‐dwelling, non‐demented older individuals from the Mayo Clinic Study of Aging, Krell‐Roesch et al. determined that lower cerebrospinal fluid (CSF) amyloid beta (Aβ)_42_, and higher total tau (t‐tau)/Aβ_42_, and phosphorylated tau (p‐tau)_181_/Aβ_42_ were associated with greater depression, anxiety, apathy, and nighttime severity.^55^ Using a shotgun proteomic analysis approach, 15 plasma proteins were associated with current and future NPS and cognitive decline, suggesting the role of other biological mechanisms.^56^

Using data from 571 non‐demented individuals from the Alzheimer's Disease Neuroimaging Initiative (ADNI) dataset, Ghahremani et al. determined that cross‐sectionally, MBI, defined as persistent NPS across two visits, was associated with higher plasma p‐tau_181_ levels cross‐sectionally, and longitudinally over 4 years.^57^ In a combined cohort from MEMENTO and ADNI, MBI was associated with lower Aβ_42/40_, higher p‐tau181, and higher t‐tau, whereas NPS–not MBI was only associated with lower Aβ_42/40_.^12^ In participants with NC or MCI from ADNI, lower Aβ_42/40_ was associated with greater severity in overall MBI and affective dysregulation.^10^ Together, these findings suggest NPS and MBI in preclinical and prodromal populations are associated with AD‐specific biomarkers of neurodegeneration, highlighting the need for further research to investigate their potential role in modulating AD risk.

Inflammatory processes have also been investigated in the context of NPS in patients with AD, which is extremely timely given the recent updates to the biological criteria for AD (ATNIVS criteria).^58^ In 87 community‐dwelling older individuals with MCI or mild dementia, NPS was associated with distinct systemic and central nervous system inflammatory processes. Specifically, NPS were associated with interleukin (IL‐6) and C‐reactive protein (CRP) in serum, and with soluble intracellular adhesion molecule‐1 (sICAM‐1), IL‐8, 10‐kDA interferon‐gamma‐induced protein, and CRP.^59^

#### 2.4.2 Neuroimaging markers

Depressive symptoms in cognitively impaired persons have been linked to reduced brain volumes implicated in AD, such as the hippocampus,^60^ medial temporal lobe, prefrontal frontal cortex, and frontal cortex.^61^, ^62^ Underlying small vessel disease, a neuroradiological finding indicating a form of leukoencephalopathy, can be detected through the quantification of white matter hyperintensities on magnetic resonance imaging (MRI). These hyperintensities have been associated with the presence and severity of delusions,^63^ and clusters of NPS such as psychosis, affective symptoms, and hyperactivity subsyndromes.^64^ However, findings with overall NPS and depression are mixed,^65^ emphasizing the need for larger validation studies that confirm the link between underlying vascular disease and NPS.

Using neuromelanin‐sensitive MRI, Cassidy et al. investigated the association between locus coeruleus (LC) integrity, a marker of norepinephrine neuron loss with AD and NPS severity. The authors reported that in AD, but not CN individuals, both LC signal and cortical tau burden independently predicted NPS severity and that their interaction was also significant, suggesting that tau may impair LC function in a disease‐specific way.^66^

In dementia‐free individuals, reduced functional connectivity between the posterior cingulate cortex and the medial prefrontal cortex was associated with the presence of MBI, supporting the involvement of the default mode network.^67^ Additionally, reduced functional connectivity between the anterior cingulate cortex and left anterior insula in the presence of MBI supported the involvement of the salience network. These findings corroborate previous structural imaging reporting associations between atrophy in the entorhinal cortex, hippocampus, parahippocampal gyrus, and temporal lobe with MBI and hippocampal atrophy with motivation and impulse dyscontrol^68^, ^69^

In CN older adults recruited from the Mayo Clinic Study of Aging, NPS synergistically interacted with cortical amyloid deposition as assessed by florbetapir (Pittsburgh compound B [PiB]) on global and domain‐specific cognitive decline, especially with regard to attention/executive function.^70^ Specifically, significant interactions were reported between PiB positron emission tomography (PET) and agitation, appetite, euphoria, irritability, anxiety, and depression. While those findings in individuals with NC support an association between Aβ and NPS on cognitive decline, Dang et al. reported that in AD patients, NPS were associated with the presence of tau pathology as assessed by 18F‐flortaucipir, but not Aβ as assessed by PiB PET.^71^ Similarly, in a study investigating the association between amyloid/tau/neurodegeneration neuroimaging markers and cognitive impairment and NPS, NPS in AD was associated with tau, followed by hypometabolism, but not with Aβ.^71^ In the TRIAD cohort (*n* = 222), NPS severity was associated with tau pathology as assessed by 18F‐MK6240, but not Aβ as assessed by 18F‐AZD4694. Furthermore, associations between NPS severity and tau PET were greater in the parietal area and superior frontal, temporal, and medial occipital lobes, which are regions vulnerable to early AD pathology and which are part of the behavioral circuits.^72^ Collectively, these findings suggest that while Aβ has a key role in the early stages of AD, tau may have a larger role in the manifestation of NPS in AD. Furthermore, tau has been linked to neuroinflammatory processes, particularly microglial activation through the cyclic GMP‐AMP synthase (cGAS)‐stimulator of interferon genes (STING) pathway,^73^ which may contribute to the expression and severity of NPS. Specifically, Schaffer et al. reported that microglial activation in the frontal, temporal, and parietal cortices were associated with NPS severity, with irritability having the greatest association.^74^ This neuroinflammatory response may mediate tau‐related effects on cognition and behavior, suggesting that tau pathology not only drives cognitive decline but also influences the neuroinflammatory pathways that underlie NPS in AD.

### 2.5 COVID‐19

Since COVID‐19 was declared a global pandemic, the World Health Organization implemented restrictions (quarantine, physical/social, and distancing), which have had profound impacts on patients with dementia and their caregivers. Specifically, individuals with dementia experienced a loss of respite and community services and reduced social support and care via hospital visits, consequently reducing the support provided for caregivers. While restrictions reduced the spread of COVID‐19, these required precautions exacerbated the behavioral and cognitive symptoms in dementia patients and also increased caregiver burden.

Wei et al. evaluated the impact of COVID‐19 and related restrictions on caregivers and people living with dementia using data collected from an international survey with respondents from Australia, Germany, Spain, and the Netherlands.^75^ Individuals with dementia experienced worsening of NPS after the outbreak of COVID‐19, specifically with regard to depression, apathy, delusions, anxiety, irritability, and agitation. The authors also reported that worsening of NPS was associated with a limited understanding of COVID‐19, and not living with a caregiver. For caregivers, worsening mental health was associated with uncertainty about the future, and loneliness. Those findings emphasize the need to consider the impacts of COVID‐19 on NPS as we move toward interpreting the evidence of studies that were conducted during the pandemic. The findings of this study were corroborated by Custodio et al., in which worsening of NPS was observed in 81% of patients with MCI or AD during the lockdown, and only 10% saw a return to baseline after the lockdown.^76^ Of importance, individual NPS that seem to be most impacted are depression, anxiety, apathy, agitation, irritability, psychosis, and sleep disturbances.^77^, ^78^

In addition to the worsening of NPS, two studies in nursing home settings reported that during the COVID pandemic, there was an increase in antipsychotic,^79^ antidepressant, antianxiety, and opioid prescriptions,^80^ highlighting the negative implications of increased isolation at the facility level, and the need for pro‐active strategies to monitor and assess symptoms to determine the need for psychoactive medication use.

Beyond the immediate effects of COVID‐19 restrictions, long COVID has emerged as a significant factor in the manifestation of novel NPS, even in individuals without dementia. These symptoms include depression, anxiety, and sleep disturbances, which persist beyond the acute phase of infection.^81^ This may be due to the persistent inflammation and immune dysregulation in those with long COVID, which has been linked to NPS presence and severity.^82^, ^83^ As such, it is essential to consider the broad impacts of COVID‐19 on brain health and behavior, as NPS related to long COVID may overlap in patients with AD and related neurodegenerative disorders.

### 2.6 Status of interventions to date

Despite the significant impact of NPS on AD outcomes, treatment options with strong efficacy and safety are limited. Non‐pharmacological interventions are first‐line treatments and have shown positive results,^84^ but their real‐world application is limited due to inconsistent caregiver training. Individuals with severe NPS are often transferred to emergency departments or hospitals, which can worsen symptoms and increase health‐care costs. Keeping individuals in familiar environments is a key clinical goal. While non‐drug interventions are preferred, emerging evidence supports pharmacological options when needed.

In 2022, Freedman et al. reported on the results of their Virtual Behavioral Medicine Program (VBM), a model of virtual care that implements both non‐pharmacological and pharmacological approaches to support the management of patients with NPS in their own environment.^85^ The authors reported that individuals referred to VBM reduced transfers to the behavioral unit by > 60%. While this program may be scalable at the international level, this study will need to be replicated in a larger sample size to confirm findings.

Oftentimes, non‐pharmacological interventions need to be supplemented with pharmacological interventions for the management of NPS. With regard to pharmacological interventions, only one medication has been approved for the management of NPS in AD in Canada and Europe: risperidone for the short‐term management of agitation. In 2022, six trials were actively investigating novel and repurposed agents for agitation in AD: two separate trials investigated synthetic cannabinoids, and one trial each investigated the use of an antidepressant (escitalopram), an atypical antipsychotic (brexipiprazole), a serotonin‐6 receptor antagonist (SUVN‐502), and the deuterated form of dextromethorphan/quinidine (AVP‐786). Details of those trials are provided in Table 2.

**TABLE 2:** Summary of ongoing clinical trials for agitation in AD.

Trial	Drug type	Mechanism of action	Sample size	Main outcome	Trial duration/Study Phase	Justification/Rationale for trial
*NCT04516057* Nabilone	Synthetic cannabinoid	Partial agonist at cannabinoid receptors 1 and 2 (CB1 and CB2)	112	CMAI	8 weeks Phase III	**Pilot study results**: Herrmann et al., AJGP 2019^42^
*NCT02792257* Dronabinol	Synthetic cannabinoid	CB1 full agonist	160	PAS	3 weeks Phase II	**Baseline data**: Cohen et al., Int Psychogeriatr 2021^86^
*NCT03108846* Escitalopram	Antidepressant	Serotonin (5‐HT) re‐uptake inhibitor	392	ADCS‐CGIC	12 weeks Phase III	**Study Design**: Ehrhardt et al., Alzheimers Dement, 2019^87^, ^88^ **CitAD trial**: Porsteinsson et al., JAMA, 2014^88^
*NCT02442765* AVP‐786	Deuterated form of Dextromethorphan/ Quinidine (AVP‐923)	NMDA antagonist and sigma 1 receptor agonist	387	CMAI	12 weeks Phase III	**Rationale**: Khoury et al., Exp Opin Pharmacother, 2021^89^
*NCT03620981* Brexpiprazole	Atypical Antipsychotic	Dopamine D1 and serotonin 1A partial agonist	410	CMAI	10 weeks Phase II/III	**First two trials**: Grossberg et al., AJGP, 2020^90^ Lee et al., JAMA Neurology, 2023^91^
*NCT05397639* SUVN‐502 Masurpirdine	–	Serotonin‐6 receptor antagonist	375	CMAI	12 weeks Phase III	**Subgroup analysis of main trial results**: Niroji et al., IJGP 2022^92^

Abbreviations: AD, Alzheimer's disease; ADCD‐CGIC, Modified Alzheimer's Disease Cooperative Study–Clinical Global Impression of Change; CMAI, Cohen Mansfield agitation inventory; PAS, Pittsburgh Agitation Scale.

A review of the updated evidence on pharmacological interventions supports the use of aripiprazole, given its demonstrated efficacy and safety in the treatment of AD‐related psychosis, with some promise for pimavanserin based on clinical trial evidence.^42^ Though there are no approved treatments for apathy to date, methylphenidate has demonstrated improvements in apathy compared to placebo in phase II clinical trials.^86^

## 3 ROAD MAP FOR FUTURE STUDIES

The presence of NPS is prevalent and has widespread negative, sustained, and often compounded impacts on patients, caregivers, and society. The association between NPS and incident AD and related outcomes emphasizes the need to improve the diagnosis, elucidate the underlying neurobiology, and identify safe and effective treatments for NPS. In an interdisciplinary effort to move this field forward, researchers have investigated and reported on a wide range of topics, including diversity and disparities, diagnostic frameworks, NPS as a non‐cognitive marker of AD, neurobiology of NPS, the impact of COVID‐19 on NPS‐related outcomes, and interventions. Research progress in these areas holds important implications for future research work and subsequent clinical implementation. Specifically, the findings of those studies structure a road map of future directions that can and should be incorporated into the design of future studies (Table 3).

**TABLE 3:** Road map for future studies.

	Health‐related disparities	Diagnostic criteria	Non‐cognitive markers of dementia	Neurobiology	COVID‐19	Clinical trials
Short term (1–5 years)	‐ Recruit multi‐ethnic cohorts from diverse geographic and socioeconomic backgrounds to study NPS as a risk factor for AD	‐ Embed existing diagnostic frameworks into observational studies and clinical trials to assess and test their utility in patient identification and treatment monitoring	‐ Conduct validation studies with non‐cognitive markers investigating their capacity to predict AD risk in patients with NPS ‐ Launch pilot studies investigating the impact of rehabilitative interventions for non‐cognitive markers (e.g., hearing aids, driving, cognitive retraining, gait) on NPS severity and AD risk	‐ Complete analyses of fluid and imaging markers associated with NPS and MBI in preclinical and prodromal AD patients ‐ Investigate the diagnostic and prognostic value of markers for risk stratification	‐ Conduct studies to assess the immediate impacts and long‐term impacts of COVID‐19 on NPS in patients and caregivers ‐ Increase public and caregiver awareness about the connection between COVID‐19 and NPS, including strategies to help reduce burden	‐ Initiate trials that integrate pharmacological and non‐pharmacological interventions, which would provide insight into their combined efficacy in managing NPS ‐ Conduct larger phase II and III trials for agents in early‐phase trials to confirm efficacy in managing specific NPS in AD
Medium term (5–10 years)	‐ Test diagnostic tools for NPS that are invariant to race/ethnicity to ensure equitable measurement across diverse groups^a^ ‐ Investigate the impact of race, sex, and SES with modifying NPS and AD neurobiological pathways ‐ Include S/SDOH outcomes in studies to investigate how it modifies NPS‐related outcomes	‐ Pair diagnostic criteria with biological markers in longitudinal studies to improve diagnostic precision and predict treatment outcomes in patients with NPS ‐ Introduce diagnostic criteria into clinical workflows to identify patients with NPS and monitor progression	‐ Extend validation studies into diverse populations to ensure the generalizability of findings ‐ Develop clinical tools that integrate non‐cognitive markers with traditional assessments of cognition and NPS	‐ Elucidate precise mechanisms linking AD and neurodegenerative pathways to NPS ‐ Design and conduct clinical trials for NPS focusing on interventions informed by biomarker data ‐ Assess the reproducibility of findings across diverse cohorts, including underrepresented populations	‐ Investigate the interplay of persistent inflammation, immune dysregulation, NPS, and neurodegenerative processes in AD patients with long COVID ‐ Establish longitudinal studies to track NPS progression in AD patients with ongoing impacts of long COVID	‐ Establish infrastructure for widespread implementation of evidence‐based non‐pharmacological and pharmacological interventions ‐ Implement studies investigating personalized approaches using both clinical and biomarker data for targeted interventions
Long term (10+ years)	‐ Confirm generalizability of findings through international and multi‐site cohort studies ‐ Develop clinical guidelines that incorporate the intersections of multiple sources of disparities to improve early diagnostics and management of NPS^b^	‐ Refine and adapt diagnostic criteria based on emerging evidence in electronic health records	‐ Achieve consensus on the use of non‐cognitive markers in clinical trials and clinical practice across various settings, including telehealth ‐ Provide definitive evidence on the efficacy of interventions for non‐cognitive markers for reducing NPS severity and AD risk	‐ Reduce health‐care disparities by expanding biomarker validation studies in NPS across diverse populations ‐ Establish precision medicine frameworks that implement biomarker data with personalizing AD interventions for NPS ‐ Use biomarkers as regulatory endpoints to support the approval of new therapies for NPS	‐ Validate and implement frameworks that combine pandemic preparedness, mental health support, and dementia care, ensuring resilience against future pandemics or similar major disruptive events	‐ Establish an integrated care framework that combines pharmacological and non‐pharmacological interventions that are tailored to an individual's needs, enabling seamless management of NPS across care settings ‐ Collaborate with policy makers to adopt evidence‐based interventions for NPS

^a,b^ can also be applied to “diagnostic criteria.”

Abbreviations: AD, Alzheimer's disease; MBI, mild behavioral impairment; NPS, neuropsychiatric symptoms; SES, socioeconomic status; S/SDOH, social and structural determinants of health.

Health‐related disparities such as race, ethnicity, sex, and gender are being increasingly acknowledged as having an essential role in AD‐related research rather than just as covariates to adjust for in models. However, a large body of evidence that guides clinical practice guidelines and diagnostic frameworks overlooks the impact of health‐related disparities. Future studies should use multi‐ethnic cohorts from diverse socioeconomic status to elucidate the impact of ethnoracial groups on NPS as a risk factor for AD. In addition, studies examining the effectiveness of other measures of biological and psychological stress or diagnostic tools of NPS that are invariant to race/ethnicity would be beneficial to the design and conduct of future research work. Of importance, social and structural determinants of health (S/SDOH)^87^ have been associated with AD,^88^ and have been reported to modify its association with race/ethnicity^89^ and sex^90^ and AD‐related biology.^91^ Therefore, it would be plausible to assume that S/SDOH would impact the association between NPS and AD‐related outcomes; however, this has not been well explored. Future studies investigating how S/SDOH impacts the association between NPS and AD are needed to ensure that individuals from differing backgrounds are equally represented in neuropsychiatric research and to confirm the generalizability of past, present, and future findings in this field. Many studies also lack longitudinal data on the association between health‐related disparities and the influence of the progression of NPS over time. Therefore, future studies should incorporate longitudinal study designs to monitor the impact of health‐related disparities on the development and progression of NPS in AD.

As NPS are often undertreated and underdiagnosed, the creation and revision of diagnostic criteria have high potential impacts on the identification of individuals in need of therapeutic intervention. However, implementing standardized diagnostic frameworks for NPS in AD into clinical practice and research can be challenging due to the variability in health‐care settings, resource constraints, and the need for specialized training. Therefore, future work should focus on developing and validating diagnostic tools that are both comprehensive and adaptable to diverse clinical environments and that can be integrated into routine clinical workflows that would enhance early detection and intervention for NPS and/or MBI in AD. Future studies are also encouraged to include the diagnostic criteria as appropriate when identifying individuals for clinical trials and research studies and monitoring of treatment response. Collectively, integrating diagnostic frameworks for NPS and MBI into clinics and research could improve diagnosticating, allowing for early treatment and monitoring, which could slow down cognitive decline, given their strong associations with AD risk.

The validation of non‐cognitive markers of dementia has high implications for patients, researchers, and clinicians, given their cost‐ and time‐effectiveness, and ability to predict dementia risk. However, the scarcity of validated non‐cognitive markers hinders early detection and intervention. Pairing non‐cognitive markers with assessments of NPS in the clinic could improve the detection of at‐risk individuals who are in need of early intervention and monitoring to slow down the progression of cognitive decline. Of importance, changes in driving, an important functional activity and independent activity of daily living (IADL), have been linked to preclinical AD and AD pathophysiology.^92^ Babulal et al. reported that in NC older individuals, those with greater NPS and abnormal CSF markers of AD pathophysiology were more likely to have their driving performance impacted.^93^ Therefore, future studies with non‐cognitive markers of dementia should also consider the evaluation of driving performance and behavior. Future studies may consider investigating whether rehabilitation of non‐cognitive markers of dementia, such as hearing loss and gait, would have improvements in NPS, functional activities, and lowering AD risk.

Elucidating the underlying neurobiology of NPS, and how this impacts AD risk, has garnered substantial interest as it provides insights into novel biological markers and mechanisms that can be targeted for therapeutic intervention. Recent evidence provides continued support for the association between AD‐specific markers and the presence and severity of NPS, reiterating that the presence of NPS is a marker of AD risk and disease progression. However, the intersection between neurobiology and health disparities and how this relates to NPS and MBI has not been thoroughly investigated. Future studies should investigate how disparities intersect with sex/gender, race/ethnicity, and socioeconomic status when investigating the neurobiology of NPS. These studies would inform how our understanding of the neurobiology of NPS is generalizable or unique based on underlying disparity, which would inform the conduct of future research studies and clinical trials, and ensure that evidence is interpreted appropriately.

COVID‐19 has undoubtedly impacted patients, caregivers, and the health‐care system. Specifically, patients have experienced increased presence and severity of NPS, which have increased the associated burden on caregivers. Furthermore, with increased psychotropic use, not only is the health‐care system experiencing an increased economic burden but patients are subjected to adverse effects associated with polypharmacy. Therefore, knowledge users are encouraged to review the evidence of studies conducted during COVID‐19 appropriately, understanding that NPS are likely more prevalent and more severe compared to studies conducted prior to COVID‐19. Of importance, the long‐term effects of COVID‐19 on the trajectory of NPS in patients with AD remain largely unexplored. Investigating the longitudinal effects of long COVID on NPS in AD is essential for informing post‐pandemic care and developing strategies that can be integrated into clinical frameworks for potential future health crises.

Recent clinical trials for the management of NPS are a necessary critical step to improving the lives of patients with underlying NPS. As currently approved pharmacological treatments for the management of NPS have high‐risk safety profiles and modest efficacy, the investigation of novel therapeutic agents addresses a high unmet clinical need for patients with NPS. In addition to studies investigating the safety and efficacy of novel agents for the management of NPS, future studies are encouraged to investigate the efficacy of non‐pharmacological intervention for NPS, which could be implemented prior to the initiation of psychotropics, or in combination with psychotropics. Future trials are also encouraged to use appropriate diagnostic tools and assessments to optimize patient enrichment and monitoring of treatment response.

## CONFLICT OF INTEREST STATEMENT

The authors report no competing interests Author disclosures are available in the supporting information.

## CONSENT STATEMENT

Consent was not necessary for this manuscript.

## Supporting information



Supporting Information
